# Supplementary material for: Early Individual and Family Predictors of Weight Trajectories From Early Childhood to Adolescence: Results From the Millennium Cohort Study
Source: Front Pediatr. 2020 Aug 11;8:417. doi: 10.3389/fped.2020.00417 (PMC7431491; doi:10.3389/fped.2020.00417)
Supplement: Supplementary file 1 [file Data_Sheet_1.pdf]

Early individual and family predictors of weight trajectories from early childhood to adolescence: results from the Millennium Cohort Study

Constança Soares dos Santos[1,2,3*], João Picoito[2,3,4], Carla Nunes[2,3], Isabel Loureiro[2,3]

[1]Department of Pediatrics, Centro Hospitalar Universitário Cova da Beira, Covilhã, Portugal
[2]Escola Nacional de Saúde Pública, Universidade NOVA de Lisboa, Lisboa, Portugal
[3]Centro de Investigação em Saúde Pública, Escola Nacional de Saúde Pública, Universidade NOVA de Lisboa, Lisboa, Portugal
[4]Department of Child and Adolescent Psychiatry, Centro Hospitalar e Universitário de Coimbra, Coimbra, Portugal, Portugal

Front. Pediatr. 8:417. doi: 10.3389/fped.2020.00417

SUPPLEMENTARY MATERIAL

Supplementary Table 1 – Child and Family covariates

	Response options	Recoded
	Sociodemographic, Economic and Cultural Context	
**Ethnicity**	White / Mixed / Indian / Pakistani and Bangladeshi / Black or Black British/ Other	White/ Other<br>Ref: (1)
**Family structure (Sweep 1)**		Two carers or parents<br>One carer or parent
**OECD Median Poverty Indicator (Sweep 1)**		Above 60%<br>Below 60%
**Mother age at cohort member birth**	Continuous (years)	<20 Y<br>20-40 Y<br>>40Y
**Mother nutritional status (Sweep 1)**	BMI (continuous)	Underweight<br>Normal weight<br>Overweight/obesity<br>Ref: (2)
**Mother education (Sweep 1)**	NVQ 1 / NVQ 2 / NVQ 3 / NVQ 4 / NVQ 5 / NVQ 6 / None / Overseas qualification	NVQ >=4<br>NVQ<4, none or overseas<br>Ref: (3)
	Perinatal and Early Infancy	
**Gestational age**	Continuous (days)	Extreme to moderate PT (<= 33w+6d)<br>Late PT (34w to 36w+6)<br>Term (>=37w)<br>Ref: (4)
**Birthweight**	Continuous (kg)	Normal birthweight (2,5-4kg)<br>Low birthweight (<2,5kg)<br>High birthweight (>4kg)<br>Ref: (5)
**Birthweight for gestational age**	Continuous (centiles)	Appropriate for GA (P10-90)<br>Small for GA (<P10)<br>Big for GA (>P90)<br>Ref: (6)

**Breastfeeding duration**	Continuous (days)	Never<br><= 2 months<br>2 to 4 months<br>4 to 6 months<br>6 to 12 months<br>>= 12 months<br>Ref: (7)
**Age at complementary feeding**	Continuous (days)	Between 4 and 6 months<br>Early introduction<br>Late introduction<br>Ref: (8)
Child temperament and self-regulation		
**Child temperament - Carey Infant Temperament Scale (Sweep 1)**		
He gets sleepy at about the same time each evening<br>His naps are about the same length from day to day<br>He wants and takes solid food at about the same time<br>He wants and takes milk feeds at about the same time	Almost never / Rarely / Usually does not / Often / Almost always	Sum score - Carey – Regularity<br>Ref: (9)
He is fretful for few minutes in a new place or situation<br>He appears bothered when put down in a different sleeping place<br>He objects to being bathed in a different place/ by a different person after 2 or 3 tries.<br>He is still wary of strangers after 15 minutes.<br>He is shy on meeting another child for the first time.	Almost never / Rarely / Usually does not / Often / Almost always	Sum score - Carey – Adaptability and Approach/ Withdrawal<br>Ref: (9)
He makes happy sounds when changing nappy or being dressed.<br>He is pleasant when arriving in unfamiliar places.<br>He is pleasant during like hair brushing or face washing.<br>He is content during interruptions of feeding.<br>He remains pleasant or calm with minor injuries.	Almost never /Rarely / Usually does not / Often / Almost always	Sum score - Carey – Mood<br>Ref: (9)
**Child self-regulation - Child Social Behaviour Scale (Sweep 1)**		
Likes to work things out for self<br>Does not need much help with tasks<br>Chooses activities on his own<br>Persists in the face of difficult tasks<br>Move to new activity after finishing task	Not true / Somewhat true / Certainly true	Average score - CSBQ – Independence and Self-regulation<br>Ref: (10)
Shows mood swings<br>Gets over excited<br>Easily frustrated<br>Gets over being upset quickly<br>Acts impulsively	Not true / Somewhat true / Certainly true	Average score – CSBQ – Emotional Dysregulation<br>Ref: (10)
General Parenting and Child-Parent Relationship		
**Parenting beliefs (Sweep 1)**		
Babies need to be stimulated to develop well.<br>Talking, even to a young baby, is important.<br>Cuddling a baby is very important.	Strongly disagree / Disagree/ Neither agree nor disagree / Agree / Strongly agree	Sum score – Parenting beliefs<br>Ref: (11)
**Routines (Sweep 2)**		
Does CM go to bed at regular times<br>Does CM have meals at regular times	Never or almost never/ Sometimes/ Usually / Always	Sum score – Routines
**Parenting activities (Sweep 2)**		
How often do you read to child<br>How often do you try to teach the alphabet<br>How often do you try to teach counting<br>How often do you teach songs/rhymes<br>How often does child paint/draw at home	Occasionally or less than once a week / 1-2 days per week / Several times a week / Everyday	Sum score – Parenting activities
**Discipline practices - Murray Straus's Conflict Tactics Scale (Sweep 2)** (alpha= 0,70)<br>**How often do you do the following when child is naughty**		
Smack<br>Shout<br>Tell off	Never / Rarely / Once a month / Once a week or more / daily	Sum score – Harsh parenting<br>Ref: (12)

Ignore<br>Send to bedroom/naughty chair<br>Take away treats	Never / Rarely / Once a month / Once a week or more / daily	Sum score – Positive parenting<br>Ref: (12)
**Child-parent relationship - Pianta Short Form (Sweep 2)**		
We share an affectionate relationship<br>Child will seek comfort from me if upset<br>Child values his/her relationship with me<br>When I praise him/her, child beams with pride<br>Child spontaneously shares information<br>It is easy to tune in to child's feelings<br>Child openly shares feelings and experiences with me	Definitely does not apply / Not really / Neutral, not sure / Applies sometimes / Definitely applies	Average score – Pianta Closeness
Child and I always seem to be struggling with each other<br>Child is uncomfortable with physical affection or touch<br>Child easily becomes angry with me<br>Child remains angry and resistant after being disciplined<br>Dealing with my child drains my energy<br>When child wakes up in a bad mood, I know we're in for a long and difficult day<br>Child feelings towards me can be unpredictable<br>Child is sneaky or manipulative with me	Definitely does not apply / Not really / Neutral, not sure / Applies sometimes / Definitely applies	Average score – Pianta Conflict

Supplementary Table 2 – Child and Family psychosocial covariates

**Child Temperament – Carey Infant Temperament Scale**						
**Carey – Regularity**	**Almost never**	**Rarely**	**Usually not**	**Often**	**Almost always**	**% missing**
Sleepy at about the same time	424 (2,7)	674 (4,2)	594 (3,7)	4285 (27)	9922 (62,4)	7,4
Naps about the same length	624 (4)	1558 (9,9)	761 (4,8)	5707 (36,1)	7139 (45,2)	8
Solid food at about the same time	344 (2,2)	652 (3,8)	557 (9,8)	4349 (27,4)	9976 (62,8)	7,5
Milk feeds about the same time	1069 (6,8)	1333 (8,5)	1142 (7,3)	4178 (26,7)	7940 (50,7)	8,8
**Carey – Adaptability**						
Fretful in a new place or situation	7335 (46,6)	4641 (29,5)	2041 (13)	1267 (8,1)	444 (2,8)	8,4
Bothered if different sleeping place	3941 (28,9)	3776 (27,7)	2564 (18,8)	2101 (15,4)	1262 (9,2)	20,5
Objects to bathing in a different place/person	6998 (59)	2233 (18,8)	1569 (13,2)	553 (4,7)	510 (4,3)	30,9
Wary of strangers	7740 (49,3)	4569 (29,1)	1793 (11,4)	1078 (6,9)	511 (3,3)	8,6
Shy on meeting another child	7700 (49,3)	3843 (24,6)	2239 (14,3)	1247 (8)	586 (3,8)	9
**Carey - Mood**						
Happy sounds when changing nappy	916 (5,8)	1163 (7,3)	1372 (8,6)	4988 (31,4)	7446 (46,9)	7,5
Pleasant in new places	309 (2)	990 (6,2)	1505 (9,5)	5982 (37,8)	7055 (44,5)	7,7
Pleasant during procedures like hair brushing or face washing.	1031 (6,5)	2114 (13,3)	2824 (17,8)	5363 (33,8)	4527 (28,5)	7,6
Content during feeding interruptions	1013 (6,4)	1911 (12,1)	2530 (16)	5518 (34,9)	4856 (30,7)	7,8
Calm with minor injuries	974 (6,5)	1911 (12,7)	2912 (19,3)	5792 (38,5)	3469 (23)	12,3
**Child Self-regulation - Child Social Behaviour Questionnaire**						
**Independence / self-regulation**	**Not true**	**Somewhat true**	**Certainly true**			
Likes to work things out for self	576 (4)	5529 (38)	8436 (58)			15,3
Doesn’t need much help with tasks	1427 (9,9)	8363 (57,9)	4655 (32,2)			15,9
Chooses activities on his own	266 (1,8)	4275 (29,3)	10041 (68,9)			15,1
Persists in difficult tasks	1387 (9,9)	7802 (55,8)	4792 (34,3)			18,6
Move to new activity after finishing task	383 (2,7)	4660 (32,4)	9335 (64,9)			16,2
**Emotional dysregulation**						
Shows mood swings	4650 (32,3)	6201 (43)	3554 (24,7)			16,1
Gets over excited	2925 (20,2)	6805 (47,1)	4729 (32,7)			15,8
Easily frustrated	4137 (28,8)	6615 (46,1)	3594 (25,1)			16,4
Gets over being upset quickly	1080 (7,4)	4939 (33,9)	8544 (58,7)			15,2
Acts impulsively	4218 (30,3)	6573 (47,3)	3115 (22,4)			19
**Parenting beliefs**						
	**Strongly disagree**	**Disagree**	**Neither agree nor disagree**	**Agree**	**Strongly agree**	
Importance of being stimulated	47 (0,3)	66 (0,4)	520 (3,3)	5053 (31,9)	10145 (64,1)	7,8
Importance of talking	38 (0,2)	6 (0,03)	37 (0,2)	2832 (17,8)	13041 (81,7)	7,1
Importance of cuddling	33 (0,2)	16 (0,1)	129 (0,8)	2525 (15,8)	13254 (83,1)	7
**Household routines**						
	**Never / almost never**	**Sometimes**	**Usually**	**Always**		
Regular bedtime	1184 (7,7)	2138 (14)	5730 (37,5)	6243 (40,8)		10,9
Regular mealtime	325 (2,1)	1175 (7,7)	6559 (42,9)	7236 (47,3)		10,9
**Parenting Activities**						
	**Occasionally/ < 1x/ week**	**1-2 days/week**	**Several times / week**	**Everyday**		
Reading	1238 (8,1)	2400 (15,7)	2893 (18,9)	8764 (57,3)		10,9

Help child learning alphabet	1894 (15,3)	3303 (26,7)	4159 (33,6)	3009 (24,3)		28
Help child learning counting	782 (5,3)	2070 (14,1)	4443 (30,2)	7398 (40,4)		14,4
Help child learning songs/rhymes	594 (4,1)	1590 (10,9)	4251 (29,2)	8105 (55,7)		15,3
Drawing	592 (4)	2173 (14,5)	5481 (36,6)	6736 (45)		12,7
**Discipline practices – Straus's Conflict Tactics Scale**						
**Harsh parenting**	**Never**	**Rarely**	**1x/ month**	**>= 1x/ week**	**Daily**	
Smack	4467 (33,6)	6933 (52,2)	642 (4,8)	1116 (8,4)	128 (1)	22,6
Shout	445 (3,4)	4341 (32,8)	987 (7,5)	5050 (38,2)	2404 (18,2)	22,9
Tell off	106 (0,8)	1675 (12,6)	593 (4,5)	5828 (43,9)	5081 (38,3)	22,6
**Positive parenting**						
Ignore	2932 (22,7)	4622 (35,7)	704 (5,4)	3144 (24,3)	1541 (11,9)	24,6
Send to bedroom/naughty chair	3353 (25,2)	3532 (26,5)	1593 (12)	3885 (29)	964 (7,2)	22,4
Take away treats	1923 (14,6)	4671 (35,6)	1691 (12,9)	4034 (30,7)	814 (6,2)	23,5
**Parent Child Relationship – Pianta Short Form**						
**Pianta - Closeness**	**Definitely doesn't apply**	**Not really**	**Neutral, not sure**	**Applies sometimes**	**Definitely applies**	
Affectionate relationship	126 (0,9)	31 (0,2)	71 (0,5)	413 (2,4)	12935 (95,3)	20,9
Seeks comfort	119 (0,9)	74 (0,5)	62 (0,5)	938 (6,9)	12422 (91,2)	20,7
Values relationship	158 (1,2)	32 (0,2)	262 (2)	556 (4,2)	12365 (92,5)	22,1
Beams with pride when praised	99 (0,7)	64 (0,5)	86 (0,6)	1191 (8,8)	12149 (89,4)	20,8
Spontaneously shares information	110 (0,8)	334 (2,5)	370 (6,1)	2838 (21,7)	9619 (72,5)	22,7
Easy to tune with child's feelings	115 (0,9)	233 (1,7)	401 (3)	2876 (21,3)	9867 (73,1)	21,4
Openly shares feelings	207 (1,5)	339 (2,5)	327 (2,4)	2467 (18,3)	10119 (75,2)	21,6
**Pianta - Conflict**						
Always struggling	7564 (55,6)	3866 (28,4)	246 (1,8)	1667 (12,3)	250 (1,8)	20,8
Uncomfortable with physical affection	12105 (89,2)	829 (6,1)	51 (0,4)	188 (1,4)	405 (3)	20,9
Easily angry	2461 (18,2)	5060 (37,3)	629 (4,6)	4582 (33,8)	827 (6,1)	21
Resistant after being disciplined	2562 (19)	5347 (39,6)	425 (3,1)	4286 (31,7)	892 (6,6)	21,3
Drains my energy	2983 (22)	4154 (30,6)	350 (2,6)	5243 (38,6)	845 (6,2)	20,9
Waking up in a bad mood means a long and difficult day	4204 (31)	5184 (38,3)	371 (2,7)	2789 (20,6)	1000 (7,4)	21,1
Unpredictable	6043 (44,8)	4632 (34,3)	499 (3,7)	1879 (13,9)	447 (3,3)	21,4
Sneaky or manipulative	7420 (55)	3946 (29,2)	369 (2,7)	11471 (10,9)	288 (2,1)	21,4

Data are given in n (%)

Supplementary Table 3 – Differences between analytic and non-analytic samples

		Sample (n=17165)	Excluded (n=2078)	Pearson Chi² *p*-value
**Gender**				
	Male	51,1	54,0	**0,014**
	Female	48,9	46,0	
**Ethnicity**				
	white	82,3	79,4	**0,001**
	other	17,7	20,6	
**Gestational age**				
	Extreme to moderate PT	2,0	2,7	0,079
	Late PT	5,5	5,0	
	Term	92,6	92,3	
**Birthweight**				
	Low	7,1	8,5	0,072
	Normal	82,2	81,5	
	High	10,7	10,1	
**Birthweight for gestational age**				
	Small	7,8	9	0,08
	Appropriate	74,5	74,4	
	Large	17,8	16,6	
**Breastfeeding**				
	Never	33,4	44,0	**0,000**
	Ever breastfed	66,6	56,0	
**Age at complementary feeding**				
	Before 4 months	31,2	31,6	0,077
	Between 4 and 6 months	65,8	64,6	
	After 6 months	3	3,8	
**Family structure (Sweep 1)**				
	Two parents/ carers	83,5	77,1	**0,000**
	One parent / carer	16,5	22,9	
**OECD Median Poverty Indicator (Sweep 1)**				
	Above 60% median	64,1	53,0	**0,000**
	Below 60% median	35,9	47,0	
**Mother age at cohort member's birth**				
	12 to 19 years	8,6	10,8	**0,004**
	20 to 39 years	89,2	87,1	
	40 plus	2,2	2,1	
**Mother nutritional status at Sweep 1**				
	Underweight	12,8	14,3	0,069
	Normal weight	48,1	49,5	
	Overweight	26	24,3	
	Obesity	13,1	11,9	
**Mother education (Sweep 1)**				
	NVQ<=3 / none/ overseas	69,9	79,0	**0,000**
	NVQ=>4	30,1	21,0	

Data are given in %

Supplementary Figure 1 - single-group models

a) intercept-only

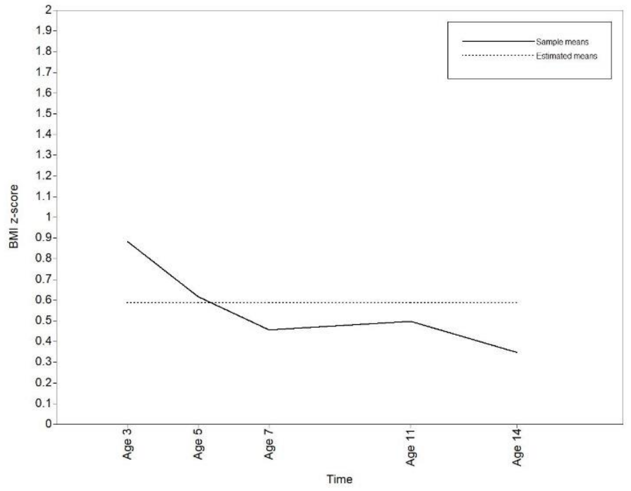

b) linear

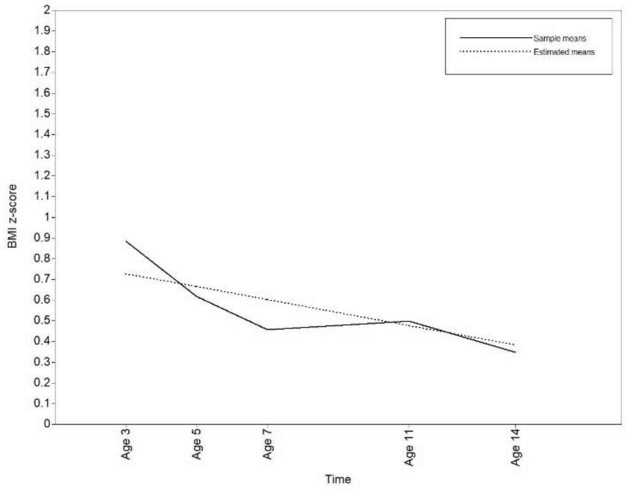

c) quadratic

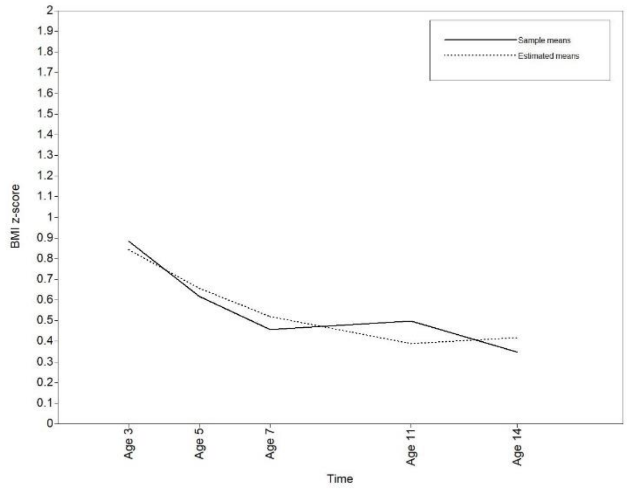

d) latent basis

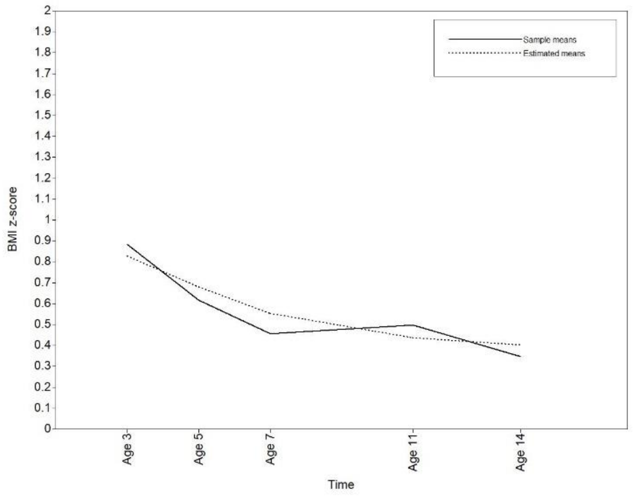

a) Intercept-only: one latent factor *intercept (i)* representing initial (baseline) levels of BMI z-scores with no further change;
b) Linear model: two latent growth factors: *intercept (i)* and *slope (s)*, representing a linear change in BMI z-scores.
c) Quadratic model: 3 latent growth factors: *intercept (i), slope (s)*, and *quadratic term (q)*, representing a quadratic change in BMI z-scores.
d) Latent basis model: 2 latent growth factor, *intercept (i)* and *slope (s)*, with loadings for the *slope* factor freely estimated (0, *, *, *, *, 1), representing the proportion of the total amount of change that has occurred up to a given point in time.

For the intercept-only model, we defined one latent factor *intercept (i)* representing initial (baseline) levels of BMI z-scores. This model showed the poorest fit of all tested models (AIC 167810, aBIC 167842). For the linear model, we defined two latent growth factors: the *intercept (i)* and the *slope (s)*, representing a linear change in BMI z-scores. This model showed a better fitting than the intercept-only model (AIC 160354, aBIC 160400)), indicating that the assumption of linear change in BMI z-scores over time is at least preferred over the assumption of no change. For the quadratic model, we added a latent growth factor *(q)* to the linear model. When estimating the quadratic model, we received a message about a negative residual variance for the Age 14

BMI z-score that was non-significant (-0,0003, *p*-value >0,05). As residuals cannot be negative, we addressed this problem by forcing this residual variance to be 0. This solution fixed the problem and no additional warnings were generated. This model showed a better fitting than the linear model (AIC 158479, aBIC 158525). For the cubic model, we added a latent growth factor *(k)* to the quadratic model. When estimating the cubic model, *(k)* and *(q)* showed a correlation of -1,005 (p-value < 0,001). As correlation cannot be equal or superior to 1, we discarded this model. Finally, we estimated a latent basis model. For this model, loadings for the *slope* factor were freely estimated (0, *, *, *, *, 1), representing the proportion of the total amount of change that has occurred up to a given point in time. This model showed a better fitting than the linear model (AIC 159506, aBIC 159566), but lower than the quadratic model.

Supplementary Table 4 - Four-Class Homoscedastic GMM - Latent Growth Factors means and variances

		Weight loss		Early Weight Gain		Early Obesity		Late Weight Gain	
			P value		P value		P value		P value
I	mean	0.723	<0,001	0.931	<0,001	3.102	<0,001	-0.716	<0,001
	variance	0.570	<0,001	0.570	<0,001	0.570	<0,001	0.570	<0,001
S	mean	-0.781	<0,001	0.428	<0,001	-0.935	<0,001	1.889	<0,001
	variance	0.500	<0,001	0.500	<0,001	0.500	<0,001	0.500	<0,001

(I) = Intercept (represents starting point); (S) = Slope (represents growth)

Supplementary Table 5.A – BMI trajectories and covariates: Unadjusted OR (n=17165)									
Variable (reference)	BMI Trajectory (reference class: Weight loss – 69%)								
	Late Weight Gain (3,3%)			Early Obesity (3,7%)			Early Weight Gain (24%)		
	OR	Lower 95%CI	Upper 95%CI	OR	Lower 95%CI	Upper 95%CI	OR	Lower 95%CI	Upper 95%CI
Male gender (female)	**0,630**	**0,434**	**0,916**	**1,688**	**1,248**	**2,282**	0,834	0,690	1,008
Ethnicity (white)	**0,139**	**0,095**	**0,203**	**0,315**	**0,224**	**0,444**	0,741	0,548	1,003
Mixed	**3,539**	**1,876**	**6,677**	**2,345**	**1,379**	**3,989**	1,416	0,891	2,250
Indian	**11,566**	**7,053**	**18,967**	1,147	0,474	2,777	0,853	0,426	1,709
Pakistani and Bangladeshi	**11,236**	**7,663**	**16,475**	**2,810**	**1,966**	**4,018**	1,310	0,917	1,872
Black and Black British	**3,802**	**2,255**	**6,409**	**7,475**	**4,985**	**11,209**	**2,073**	**1,325**	**3,242**
Other	**4,364**	**2,282**	**8,344**	**2,487**	**1,250**	**4,951**	0,852	0,381	1,901
Single-parenthood (living with both parents)	**1,895**	**1,215**	**2,957**	**1,772**	**1,194**	**2,629**	**1,421**	**1,111**	**1,819**
OECD Poverty Indicator (above 60%)	**2,248**	**1,501**	**3,365**	**1,747**	**1,302**	**2,344**	**1,669**	**1,371**	**2,032**
Mother age at cohort member birth (20-40Y)									
<20Y	0,822	0,380	1,778	0,941	0,524	1,688	0,905	0,608	1,348
>40Y	1,495	0,512	4,368	1,613	0,695	3,743	1,301	0,681	2,487
Maternal Nutritional Status (normal weight)									
Overweight/obesity	**1,722**	**1,064**	**2,789**	**4,938**	**3,448**	**7,071**	**4,233**	**3,321**	**5,395**
Underweight/obesity	1,166	0,652	2,082	0,679	0,295	1,564	0,155	0,010	2,408
High maternal education (low/none)	0,632	0,389	1,024	**0,531**	**0,359**	**0,787**	**0,578**	**0,458**	**0,729**
Gestational age (term)									
Extreme to moderate Prematurity	**3,137**	**1,408**	**6,989**	0,478	0,082	2,766	1,090	0,503	2,363
Late prematurity	1,755	0,932	3,308	1,698	0,964	2,990	**1,798**	**1,166**	**2,774**
Birthweight (normal)									
Low birthweight	**2,594**	**1,582**	**4,254**	0,748	0,370	1,516	1,006	0,650	1,558
High birthweight	0,258	0,037	1,781	**1,851**	**1,311**	**2,614**	**1,621**	**1,236**	**2,127**
Birthweight for gestational age (appropriate)									
Small for GA	**2,648**	**1,670**	**4,198**	0,802	0,395	1,626	0,870	0,568	1,332
Large for GA	0,472	0,196	1,141	**1,963**	**1,448**	**2,661**	**1,408**	**1,123**	**1,767**
Breastfeeding duration (never)									
<= 2 months	0,741	0,445	1,236	0,922	0,637	1,336	1,034	0,804	1,328
2-4 months	0,792	0,401	1,563	0,855	0,552	1,326	**0,549**	**0,353**	**0,855**
4-6 months	0,588	0,248	1,393	**0,452**	**0,223**	**0,917**	**0,571**	**0,357**	**0,915**
6-12 months	0,906	0,482	1,700	**0,406**	**0,213**	**0,773**	**0,697**	**0,492**	**0,989**
>=12 minths	1,250	0,715	2,185	0,581	0,290	1,164	**0,486**	**0,303**	**0,780**
Introduction to solid food (4-6 months)									
Early introduction	0,932	0,593	1,465	**1,921**	**1,398**	**2,640**	**1,483**	**1,198**	**1,836**
Late introduction	1,981	0,905	4,337	1,469	0,685	3,150	1,320	0,730	2,385
Carey – Mood	0,955	0,898	1,016	1,014	0,965	1,066	**1,057**	**1,021**	**1,093**
Carey – Regularity	**1,065**	**1,008**	**1,125**	1,031	0,986	1,078	1,003	0,971	1,037
Carey Adaptability	**0,892**	**0,834**	**0,953**	**0,924**	**0,887**	**0,963**	**0,966**	**0,936**	**0,997**
CSBQ - independence	0,686	0,365	1,289	0,658	0,423	1,026	1,144	0,851	1,537
CSBQ – emotional dysregulation	**1,586**	**1,104**	**2,278**	**1,678**	**1,249**	**2,253**	**1,277**	**1,029**	**1,585**

Household routines	**0,724**	**0,626**	**0,837**	**0,782**	**0,699**	**0,875**	**0,846**	**0,784**	**0,913**
Parenting beliefs	**0,859**	**0,779**	**0,948**	0,905	0,794	1,031	1,022	0,928	1,126
Parenting activities	1,032	0,991	1,075	0,997	0,966	1,029	1,005	0,980	1,031
Harsh parenting	1,118	0,947	1,320	1,100	0,949	1,275	1,075	0,981	1,178
Positive parenting	**0,924**	**0,858**	**0,996**	0,979	0,918	1,046	0,960	0,920	1,002
Pianta – Closeness	**0,916**	**0,870**	**0,964**	0,966	0,918	1,018	0,979	0,937	1,024
Pianta - Conflict	**1,040**	**1,003**	**1,079**	**1,040**	**1,013**	**1,067**	1,007	0,989	1,026

Supplementary Table 5.B – BMI trajectories and covariates: Adjusted OR (n=17165)									
Variable (reference)	BMI Trajectory (reference class: Weight loss – 69%)								
	Late Weight Gain (3,3%)			Early Obesity (3,7%)			Early Weight Gain (24%)		
	OR	Lower 95%CI	Upper 95%CI	OR	Lower 95%CI	Upper 95%CI	OR	Lower 95%CI	Upper 95%CI
Male gender (female)	0,713	0,451	1,125	**1,436**	**1,030**	**2,001**	0,781	0,606	1,006
Ethnicity (white)									
Mixed	**2,601**	**1,052**	**6,431**	**2,786**	**1,435**	**5,411**	1,564	0,833	2,935
Indian	**10,344**	**4,737**	**22,587**	1,776	0,644	4,895	0,863	0,249	2,999
Pakistani and Bangladeshi	**8,455**	**4,211**	**16,975**	**2,803**	**1,580**	**4,972**	1,272	0,755	2,144
Black and Black British	**2,877**	**1,239**	**6,680**	**8,301**	**4,312**	**15,983**	**1,970**	**0,885**	**4,385**
Other	**4,043**	**1,555**	**10,508**	**3,125**	**1,136**	**8,591**	0,866	0,213	3,516
Single-parenthood (living with both parents)	1,733	0,974	3,084	1,306	0,761	2,242	1,200	0,824	1,748
OECD Poverty Indicator (above 60%)	0,961	0,546	1,691	1,060	0,721	1,558	**1,454**	**1,061**	**1,992**
Mother age at cohort member birth (20-40Y)									
<20Y	0,666	0,277	1,602	0,604	0,284	1,286	0,610	0,345	1,078
>40Y	1,645	0,443	6,103	1,567	0,621	3,951	1,309	0,469	3,656
Maternal Nutritional Status (normal weight)									
Overweight/obesity	1,459	0,857	2,484	**4,353**	**2,922**	**6,485**	**3,978**	**3,067**	**5,160**
Underweight/obesity	0,857	0,452	1,622	0,649	0,270	1,562	**0,181**	**0,034**	**0,968**
High maternal education (low/none)	0,856	0,467	1,569	0,673	0,422	1,074	0,760	0,559	1,033
Gestational age (term)									
Extreme to moderate Prematurity	**3,354**	**1,269**	**8,866**	0,907	0,066	12,377	1,726	0,627	4,754
Late prematurity	1,895	0,949	3,785	2,203	0,977	4,964	**2,365**	**1,290**	**4,337**
Birthweight (normal)									
Low birthweight	**0,907**	**0,375**	**2,193**	0,544	0,144	2,063	0,636	0,300	1,349
High birthweight	0,513	0,081	3,227	**1,216**	**0,680**	**2,172**	**1,614**	**1,020**	**2,553**
Birthweight for gestational age (appropriate)									
Small for GA	**1,937**	**0,946**	**3,968**	0,700	0,318	1,539	0,892	0,501	1,588
Large for GA	**0,681**	**0,248**	**1,870**	**1,682**	**1,046**	**2,703**	0,940	0,637	1,387
Breastfeeding duration (never)									
<= 2 months	0,710	0,408	1,233	0,940	0,608	1,454	1,046	0,765	1,431
2-4 months	0,828	0,378	1,813	0,885	0,538	1,456	0,649	0,402	1,048
4-6 months	0,734	0,265	2,032	0,552	0,280	1,085	0,679	0,378	1,220
6-12 months	1,210	0,572	2,561	0,524	0,275	1,001	0,891	0,562	1,411
>=12 minths	1,346	0,675	2,686	0,591	0,273	1,279	0,578	0,319	1,047
Introduction to solid food (4-6 months)									
Early introduction	1,318	0,782	2,222	**1,827**	**1,275**	**2,619**	1,307	0,998	1,712
Late introduction	1,194	0,528	2,700	0,848	0,304	2,361	1,076	0,536	2,158
Carey – Mood	0,991	0,928	1,059	1,058	0,998	1,121	**1,047**	**1,006**	**1,089**
Carey – Regularity	0,979	0,918	1,044	1,001	0,952	1,053	1,007	0,969	1,046
Carey Adaptability	0,983	0,897	1,077	0,978	0,929	1,031	0,998	0,957	1,039
CSBQ - independence	1,049	0,579	1,901	0,826	0,538	1,267	1,121	0,798	1,574
CSBQ – emotional dysregulation	1,091	0,704	1,691	1,289	0,896	1,856	1,197	0,898	1,596

Household routines	**0,804**	**0,686**	**0,943**	**0,871**	**0,763**	**0,994**	**0,899**	**0,821**	**0,985**
Parenting beliefs	0,964	0,825	1,126	1,015	0,864	1,192	1,086	0,977	1,207
Parenting activities	1,018	0,976	1,062	0,999	0,962	1,037	1,005	0,974	1,036
Harsh parenting	0,957	0,856	1,069	1,026	0,943	1,116	1,059	0,991	1,132
Positive parenting	0,959	0,880	1,044	0,969	0,899	1,045	0,945	0,893	1,000
Pianta – Closeness	0,947	0,878	1,022	1,046	0,972	1,126	1,028	0,964	1,095
Pianta - Conflict	1,032	0,986	1,081	1,031	0,999	1,064	1,003	0,976	1,031

References

1. Stuart B, Panico L. Early-childhood BMI trajectories: evidence from a prospective, nationally representative British cohort study. Nutr Diabetes. 2016 Mar 7;6:e198.

2. World Health Oranization. Obesity: preventing and managing the global epidemic: report of a WHO consultation. Geneva: WHO; 2000 p. 152. Report No.: 894.

3. Flouri E, Ioakeimidi S, Midouhas E, Ploubidis GB. Maternal psychological distress and child decision-making. J Affect Disord. 2017 Aug 15;218:35–40.

4. March of Dimes, PMNCH, Save the children, WHO. Born Too Soon: The Global action report on preterm Birth [Internet]. Geneva: CP Howson, MV Kinney, Jelawn. World Health Organization.; 2012 [cited 2019 Oct 25]. Available from: https://www.who.int/maternal_child_adolescent/documents/born_too_soon/en/

5. World Health Organization. ICD-10 : international statistical classification of diseases and related health problems : tenth revision. World Health Organization; 2004.

6. Villar J, Cheikh Ismail L, Victora CG, Ohuma EO, Bertino E, Altman DG, et al. International standards for newborn weight, length, and head circumference by gestational age and sex: the Newborn Cross-Sectional Study of the INTERGROWTH-21st Project. Lancet. 2014 Sep 6;384(9946):857–68.

7. Carling SJ, Demment MM, Kjolhede CL, Olson CM. Breastfeeding duration and weight gain trajectory in infancy. Pediatrics. 2015 Jan;135(1):111–9.

8. Fewtrell M, Bronsky J, Campoy C, Domellöf M, Embleton N, Fidler Mis N, et al. Complementary feeding: A position paper by the european society for paediatric gastroenterology, hepatology, and nutrition (ESPGHAN) committee on nutrition. J Pediatr Gastroenterol Nutr. 2017;64(1):119–32.

9. Hernández-Alava M, Popli G. Children’s development and parental input: evidence from the UK millennium cohort study. Demography. 2017;54(2):485–511.

10. Anderson SE, Sacker A, Whitaker RC, Kelly Y. Self-regulation and household routines at age three and obesity at age eleven: longitudinal analysis of the UK Millennium Cohort Study. Int J Obes. 2017 Apr 24;41(10):1459–66.

11. Kroll ME, Carson C, Redshaw M, Quigley MA. Early father involvement and subsequent child behaviour at ages 3, 5 and 7 years: prospective analysis of the UK millennium cohort study. PLoS One. 2016 Sep 21;11(9):e0162339.

12. Rajyaguru P, Moran P, Cordero M, Pearson R. Disciplinary parenting practice and child mental health: evidence from the UK millennium cohort study. J Am Acad Child Adolesc Psychiatry. 2019;58(1):108–116.e2.
